# Suicidal ideation, distress, and related factors in a population of cancer patients treated in a general acute hospital

**DOI:** 10.1007/s00520-021-06429-w

**Published:** 2021-07-29

**Authors:** Bianca Senf, Bernd Bender, Jens Fettel

**Affiliations:** Department of Psycho-Oncology, University Cancer Center (UCT), Johann Wolfgang Goethe University, Theodor-Stern-Kai 7, 60590 Frankfurt/Main, Germany

**Keywords:** Cancer, Distress, Screening, Suicidal ideation, Suicide

## Abstract

**Purpose:**

Suicidality and suicidal ideation (SI) in oncology has long been an underestimated danger. Although there are cancer-specific distress screening tools available, none of these specifically incorporates items for SI. We examined the prevalence of SI in cancer patients, investigated the relation between SI and distress, and tried to identify additional associated factors.

**Methods:**

A cross-sectional study with patients treated for cancer in a primary care hospital was conducted. Psychosocial distress and SI in 226 patients was assessed. An expert rating scale (PO-Bado-SF) and a self-assessment instrument (QSC-R23) were used to measure distress. SI was assessed with item 9 of the PHQ-9. Data was descriptively analyzed, and correlations and group comparisons between clinically distressed and non-distressed patients were calculated.

**Results:**

SI was reported by 15% of patients. Classified as clinically distressed were 24.8% (QSC-R23) to 36.7% (PO-Bado-SF). SI was correlated with externally (*r*_*τ*_ = 0.19, *p* < 0.001) and self-rated distress (*r*_*τ*_ = 0.31, *p* < 0.001). Symptoms sufficiently severe for at least a medium major depressive episode were recorded in 23.5% of patients (PHQ-9). Factors associated with SI were feeling bad about oneself, feeling down, depressed, and hopeless, deficits in activities of daily life, psycho-somatic afflictions, social restrictions, and restrictions in daily life. Being in a steady relationship seemed to have a protective effect.

**Conclusions:**

SI is common in cancer patients. Distress and associated factors are increased in patients with SI. A distress screening with the ability to assess SI could be an important step in prevention, but more research is necessary.

**Supplementary Information:**

The online version contains supplementary material available at 10.1007/s00520-021-06429-w.

## Introduction

Numerous studies show a higher prevalence of suicides in cancer patients compared to the general population [1, 2]. Suicidal ideation (SI) is also elevated in cancer patients. Although suicidal thoughts in cancer patients can have different functions throughout the disease trajectory [3, 4], they are still an important predictor of suicidality [5]. A study by Brown et al. [6] showed that even passive suicidal thoughts, like a wish to die, can result in a six times elevated risk of suicide. Therefore, identifying patients having suicidal thoughts with the help of a screening procedure can be a starting point for prevention [7, 8]. Studies looking at SI in cancer patients found that factors like poor physical health, chronic medical conditions [9], and especially symptoms of demoralization [10] were associated with SI. Demoralization is a construct that combines symptoms of distress, as described by the NCCN clinical practice guidelines in oncology [11], and the feeling of subjective incompetence which results in the inability to cope with external stressors, for example a cancer diagnosis [12]. Studies have shown its usefulness in populations with somatic illnesses, especially cancer, where it showed superior predictive power compared to descriptions of distress in terms of psychiatric disorders [13]. Therefore, demoralization is an important construct in better understanding the link between distress and SI and may enhance distress screening from a suicide prevention perspective. In oncology, there is no cancer-specific distress screening tool that incorporates suicidality, but a number of studies have shown the usefulness of the PHQ-9 and its item 9 in screening for suicidality in different populations [14, 15]. For example, Walker et al. [7] found that a higher score on the PHQ-9 item 9 is associated with a higher likelihood of being suicidal in a subsequent clinical interview. Twenty-three percent of patients who answered that they had SI only half of the days were found to be suicidal in the clinical interview. Similarly, Louzon et al. [15] not only showed that PHQ-9 item 9 was able to assess SI, but also that the risk of suicide increased with the frequency of suicidal thoughts (75% increased risk when answering half of the days and up to 185% when answering nearly every day). Furthermore, there is also evidence for the relation between psychic distress and SI [10] and the endorsement of PHQ-9 item 9 in particular [16], which additionally hints at the importance of screening for distress in the context of suicide prevention. On the other hand, there are also several studies that shed doubt on the utility of PHQ-9 item 9 for identifying patients at risk for suicide [17, 18]. These studies also suggest that supplementing item 9 with other screening measures may improve its usefulness in screening for suicide. Using screening instruments that assess population-specific risk factors rather than general ones might help increasing their sensitivity and specificity and simultaneously make them more parsimonious, as a study by Senf et al. [19] suggests. This is a factor to be considered when deciding on the optimal screening tool in a certain health care context or for a certain population.

The aim of this study was therefore: First, to assess the prevalence of SI in a population of patients treated for cancer in a primary care hospital. Second, to investigate the associations between distress, assessed by two different screening tools (self and external assessment), and SI. Third, to identify additional factors associated with SI (distress-related and non-distress-related).

## Material and methods

### Participants

Participants were patients treated for cancer in a general acute hospital in Frankfurt, Germany (Markus-Krankenhaus) during a 6-month period in 2007 [20]. Included were all patients fulfilling the following criteria: minimum age of 18, sufficient command of German, no cognitive impairments, no pain or nausea, no medication influencing mental or cognitive abilities at the time of the interview, written agreement to participate in the study after obtaining informed consent. All patients had been informed by their physicians about their cancer diagnosis prior to obtaining informed consent.

### Procedures

All eligible cancer patients were approached by members of the department of psycho-oncology within the first 3 days of their stay and received extensive information about the aims, procedure, data collection, and possible risks and side effects of the study. Written informed consent was only obtained after sufficient time for deliberation (minimum 24 h) and answering possible questions to the patient’s satisfaction. Trained interviewers administered the psycho-oncological basic documentation short form (PO-Bado-SF) semi-structured interview and collected demographic data. Finally, participants were provided with the self-assessment instruments and an anonymous envelope in which they could be returned.

### Study measures

#### PO-Bado-SF

The PO-Bado-SF is a distress screening instrument, developed specifically for the use with cancer patients. It is administered in form of a semi-structured interview, which takes about 5–10 min and assesses psychosocial distress with six items on two dimensions: physical and mental. Each item is rated on a 5-stage Likert scale ranging from 0 = not stressful to 4 = very stressful [21]. A sum score > 9 has been recommended as cutoff indicating further psycho-oncological treatment [22].

#### QSC-R23

The revised version of the questionnaire on distress in cancer patients is a cancer-specific self-assessment tool. Patients rate the presence and severity of distress assessed with 23 items on a 6-step Likert scale from 0 = not applicable, 1 = applies and hardly distresses me, to 5 = applies and distresses me severely. Self-assessment with the QSC-R23 takes approximately 10 min. The 23 items can be grouped in 5 scales: psycho-somatic afflictions, anxiety, information deficits, restrictions in daily life, and social restrictions. The authors suggest a sum score of > 34 as cut-off value for an indication for further psycho-oncological treatment [23].

#### PHQ-9

The depression module of the patient health questionnaire (PHQ) is a non-cancer-specific self-rating-tool for the assessment of the nine DSM-IV criteria for major depressive disorder. A sum score of ≥ 10 was considered indicative of fulfilling the criteria of at least a moderate major depressive episode [24]. Item 9 aims at measuring SI with the question: “Over the last 2 weeks, how often have you been bothered by the following problem: thoughts that you would be better off dead, or of hurting yourself in some way?” Answers can be given on a 4-stage Likert scale ranging from 0 = not at all, 1 = several days, 2 = half the days, to 3 = nearly every day [25].

### Statistical analyses

Statistical analyses were conducted using IBM SPSS 24 software package (IBM SPSS Inc., Chicago, Illinois, USA). All statistical tests were 2-sided and *p*-values < 0.05 were considered statistically significant. Data were descriptively analyzed calculating means, standard deviations, and absolute and relative frequencies. Bivariate associations were explored by either using cross tables or calculating Chi^2^ tests as well as Phi- and Cramér’s *V*-coefficients, point-biserial-correlation-coefficients, or Kendall-Tau-b correlations. Effect sizes for the Cramér’s *V-*coefficient follow the convention by Cohen [26, 27] small effect: *V* = 0.1, medium effect: *V* = 0.3, strong effect: *V* = 0.5. Kendall-Tau-b (*r*_*τ*_) effect sizes follow the convention of Kühnel and Krebs [28] with no association: 0.00 < *r*_*τ*_ < 0.05, small association: 0.05 < *r*_*τ*_ < 0.20, medium association: 0.20 < *r*_*τ*_ < 0.50, strong association: 0.50 < *r*_*τ*_ < 0.70, very strong association: *r*_*τ*_ > 0.70. Differences between groups were investigated with the *t*-test for metric and the Mann–Whitney-*U*-test categorical variables.

## Results

### Sample

Overall, *N* = 478 patients were recruited, of those *n* = 226 (47%) had complete information in the instruments considered in this study. The average age was 59.8 years (SD = 12.4) and *n* = 142 (63%) were female. Nearly half (44.7%) of patients suffered from breast cancer, 15.5% had prostate or testicular cancer, 11.9% colon or rectum cancer, 8.8% stomach, esophageal or pancreatic cancer, other urological cancers had 6.2%, 5.8% had lung cancer, 4.0% other gynecological tumors, 0.4% hematological malignancies, and 2.7% other forms of cancer. For more information, see Table 1. The *n* = 266 patients who were included in the study were on average younger (*t*_(476)_ = 4.49, *p* < 0.001).

**Table 1:** Sociodemographic data of the participants

	%	*N*	*M*	*SD*	Min	Max
Age			59.8	12.4	18	87
Sex						
Female	62.8	142				
Male	37.2	84				
Relationship						
Steady relationship	75.7	171				
No relationship	24.3	55				
Children^a^	67.3	152	1.9	1.3	1	12
Job situation						
Employed	21.4	48				
Household chores	2.7	6				
On sick leave	19.6	44				
Unemployed	1.8	4				
Pension	49.1	110				
Other	5.4	12				
Tumor diagnosis/localization						
Mamma	44.7	101				
Gynecological tumors	4.0	9				
Lung/bronchia	5.8	13				
Prostate/testicle	15.5	35				
Colon/rectum	11.9	27				
Hematological disease	0.4	1				
Urological tumors	6.2	14				
Stomach, esophagus, pancreas	8.8	20				
Other	2.7	6				
Metastases						
Yes	17.3	38				
No	42.7	94				
Not known	40.0	88				
Current disease status						
First disease	86.0	191				
Recurrence	6.3	14				
Secondary tumor	7.7	17				
Psychopharmaceuticals/opiates						
Yes	38.5	87				
No	61.1	138				
Not known	0.4	1				

^a^Children: % and *N* refer to whether participants have children.

*M*, SD, min, and max refer to the number of children.

### Prevalence of SI

The overall prevalence of SI was 15.0%. 11.9% of patients reported SI on several days over the last 2 weeks, 1.3% on more than half of the days, and 1.8% on almost every day.

### SI and distress

Descriptive statistics for the PO-Bado-SF, QSC-R23, and PHQ-9 are shown in Table 2. Descriptive statistics for single items of all instruments can be found in table S1. Correlations between PHQ-9 item 9 and distress measures are shown in Table 3.

**Table 2:** Descriptive statistics of psychosocial distress (PO-Bado-SF, PHQ-9, QSC-R23)

	*N*	*M*	*SD*	Cut-off value	*n* (%) above cutoff
PO-Bado-SF sum score	226	7.9	4.3	> 9	36.7
PO-Bado-SF mean index	226	1.5	0.8		
QSC-R23 sum score	226	22.6	18.6	> 34	24.8
QSC-R23 mean index	226	1.0	0.8		
QSC-R23 psycho-somatic afflictions mean index	226	1.2	1.1		
QSC-R23 anxiety mean index	226	1.6	1.3		
QSC-R23 information deficits mean index	226	0.7	1.0		
QSC-R23 restrictions in daily life mean index	226	1.1	1.1		
QSC-R23 social restrictions mean index	226	0.4	0.6		
PHQ-9 sum score^a^	226	6.1	5.1	< 5 5–9 > 10	47.8
					28.8
					23.5
PHQ-9 mean index^a^	226	0.7	0.6		

^a^The PO-Bado-SF sum score and mean index are calculated on the one hand from the patients’ external assessments of all 6 items (*n* = 95) and on the other hand from the patients’ assessments of 5 (*n* = 226) of the 6 items.

**Table 3:** Correlations (Kendall-Tau-b) between PHQ-9 item 9 (“Thoughts that you would be better off dead or of hurting yourself in some way”) and PO-Bado-SF (single items and sum score), QSC-R23 (sub scales and sum score), and PHQ (single items and sum score)

			PHQ-9 item 9
PO-Bado-SF			
Fatigue/tiredness		0.13*^a^	
Functional limitations in daily livings		0.18**	
Mood swings		0.09	
Anxiety		0.14*	
Sadness		0.23**	
Other problems		0.10	
Sum score		0.19**	
QSC-R23			
Psycho-somatic afflictions		0.301** ^a^	
Social restrictions		0.278**	
Anxiety		0.191**	
Restrictions in daily life		0.259**	
Information deficits		0.277**	
Sum score		0.302**	
PHQ-9			
Little interest or pleasure in doing things	0.173** ^a^		
Feeling down, depressed, or hopeless	0.311**		
Trouble falling or staying asleep, or sleeping too much	0.278**		
Feeling tired or having little energy	0.225**		
Poor appetite or overeating	0.286**		
Feeling bad about yourself—or that you are a failure or have let yourself or your family down	0.348**		
Trouble concentrating on things, such as reading the newspaper or watching television	0.213**		
Moving or speaking so slowly that other people could have noticed? Or the opposite—being so fidgety or restless that you have been that you have been moving around a lot more than usual	0.250**		
Total score ^b^	0.318**		

^a^Classification of correlation coefficients (based on Pearson’s r coefficient and adopted analogously for Kendall-Tau-b): No correlation: 0.00 < *r*_*τ*_ < 0.05, low correlation: 0.05 < *r*_*τ*_ < 0.20, medium correlation: 0.20 < *r*_*τ*_ < 0.50, high correlation: 0.50 < *r*_*τ*_ < 0.70, very high correlation: *r*_*τ*_ > 0.70 [28]

^b^Total score of PHQ-9 calculated without item 9 (“Thoughts that you would be better off dead or of hurting yourself in some way”).

*p < 0.05.

**p < 0.01.

We found a medium to large correlation between the PO-Bado-SF and the QSC-R23 sum scores (*r*_*τ*_ = 0.46, *p* < 0.001).

When externally assessed with the PO-Bado-SF, the mean distress score was 7.9 (SD = 4.3). SI correlated weakly with externally assessed distress (*r*_*τ*_ = 0.19, *p* < 0.001). An indication for further psycho-oncological treatment (cutoff > 9) had 36.7% of patients. Subjectively, i.e., independently of the cut-off-value, the raters saw indications for further psycho-oncological treatment in 55.0% of patients. We found weak associations of SI with subjectively assessed indication for further psycho-oncological treatment (*χ*^2^ = 15.33, *V* = 0.26, *p* = 0.002) as well as with having an indication by reaching the cutoff (*χ*^2^ = 11.97, *V* = 0.23, *p* = 0.007). Furthermore, patients scoring above the PO-Bado-SF cutoff scored significantly higher in SI than patients below the cutoff (*U* = 6568.5, *p* = 0.031). See also tables S2 and S3 in the supplement.

The mean value of self-assessed distress with the QSC-R23 was 22.6 (SD = 18.6). There was a correlation of medium size between SI and self-assessed distress (*r*_*τ*_ = 0.31, *p* < 0.001). The cut-off value (> 34) was exceeded by 24.8% of patients. Thereby, those patients had an indication for further psycho-oncological treatment. An association of medium strength was found between SI and having an indication for further psycho-oncological treatment (*χ*^2^ = 25.70, *V* = 0.337, *p* < 0.001). Here, also patients scoring above the cutoff differed significantly in SI from those scoring below the cutoff (*U* = 6083.5, *p* < 0.001).

The mean value of the PHQ-9 was 6.1 (SD = 5.1). We also found a correlation of SI with the remaining symptoms of a major depressive episode (MDE; PHQ-9 without item 9: *r*_*τ*_ = 0.32, *p* < 0.001). About a quarter (23.5%) of patients scored ten or more points in the PHQ-9 and thereby had symptoms sufficiently severe for at least a medium major depressive episode. Between SI and having symptoms indicative of an MDE, we found an association of medium strength (*χ*^2^ = 42.26, V = 0.31, *p* < 0.001). Feeling bad about oneself was the item correlating most strongly with SI (*r*_*τ*_ = 0.35, *p* < 0.001), followed by feeling down, depressed, and hopeless (*r*_*τ*_ = 0.31, *p* < 0.001). Additionally, we found medium to strong correlations between feeling down, depressed, and hopeless and measures of distress (PO-Bado-SF: *r*_*τ*_ = 0.44, *p* < 0.001; QSC-R23: *r*_*τ*_ = 0.50, *p* < 0.001).

### SI and other related factors

There were also associations between the individual distress factors and SI. The item of the PO-Bado-SF most strongly associated with SI was sadness (*r*_*τ*_ = 0.23, *p* < 0.001) followed by deficits in activities of daily life (*r*_*τ*_ = 0.18, *p* = 0.002). The QSC-R23 scale psycho-somatic afflictions correlated most strongly with SI (*r*_*τ*_ = 0.30, *p* < 0.001), followed by social restrictions (*r*_*τ*_ = 0.28, *p* < 0.001), and restrictions in daily life (*r*_*τ*_ = 0.26, *p* < 0.001).

Finally, we found associations between SI and demographic variables (table S3). Being in a steady relationship (*χ*^2^ = 8.12, *V* = 0.190, *p* = 0.044), having children (*χ*^2^ = 10.10, *V* = 0.211, *p* = 0.018), and the current functional status (*χ*^2^ = 19.05, *V* = 0.230, *p* = 0.025) had medium effect strengths.

## Discussion

In this study we investigated the prevalence of suicidal thoughts as measured by item 9 of the PHQ-9 in cancer patients who were hospitalized in a primary care hospital. Additionally, we looked at the relation between SI and distress assessed by self-assessment as well as external assessment instruments and tried to identify factors associated with SI in cancer patients.

## Clinical implications

### Prevalence of suicidal ideation

Our findings show that a sizable portion of cancer patients (15%) has had suicidal thoughts at least on several days over the last 2 weeks. This is comparable to the results of other studies that employed similar assessment methods in comparable populations. For example, Zhong et al. [29] who also looked at hospitalized patients and used a single-item assessment tool found that 15.3% of patients had suicidal thoughts in the past month. Likewise, Moreno-Montoya et al. [30] reported a prevalence of SI of 13.6% in hospitalized cancer patients. Similar percentages among different populations of cancer patients are reported in various studies [31, 32]. In comparison to the general population, not only are suicide rates higher in cancer patients, but also the prevalence of SI is elevated. For example, in Germany, the prevalence of SI in the general population is 8.0% [33] and 2.0% worldwide [34]. This underscores the fact that a cancer diagnosis constitutes a major risk factor for suicidality. Yet, a recent meta-analysis shows that the prevalence of SI in cancer patients is subject to wide variation [35]. The authors report prevalence rates ranging from 0.7to 46.7%. An older review reports an even bigger variety of SI ranging from 0.8 to 71.4% [36]. This wide span is likely due to population characteristics and variation in assessment methods [35, 36]. Another factor might be the transient nature of suicidality and SI making it more comparable to a state rather than a trait [5]. This is also exemplified by the fact that suicidal thoughts and behaviors can be observed during all stages of the disease trajectory in different populations [3, 4, 9, 37]. In our opinion, this highlights two crucial points. First, a better understanding of the interactions between SI and completed suicides in cancer patients is necessary. Up to now, there is little understanding of the timing of suicide risk and its associated factors and it remains unclear whether findings are consistent across different populations. A meta-analysis planned by Calati et al. [38] will address these issues. This also suggests that more research especially focusing on longitudinal designs is needed. Second, it highlights the need for health professionals to be aware of the fact that cancer patients are a risk population and that therefore the necessary precautions should be taken. A screening for suicidality would be one such measure. Yet, especially in acute settings, resources are scarce and the implementation of screening procedures is often met with resistance. For example, in Germany, certification as an oncological center or organ center by the German Cancer Society requires the establishment of a distress screening, which is often met with numerous barriers [39]. This makes the implementation of an additional screening seem unfeasible. Therefore, it should be further investigated whether established distress screenings are able to also identify patients at risk for suicidality. Lastly, education of health care workers concerning suicidality and associated risk factors in cancer patients is the essential basis for any prevention strategies [40] and is a desire expressed by health care workers themselves [41].

### Suicidal ideation and psychosocial distress

In our study, depending on the instrument, a quarter to a third of patients were clinically distressed (i.e., scoring above the respective cutoff). Clinically distressed patients agreed significantly more often with item 9 than non-distressed patients. This holds for all distress screening tools. Patients who scored above the cutoff (≥ 10) in the PHQ-9 and thus can be classified as showing symptoms of at least moderate major depressive episode [16, 24] also differed significantly from patients below the cutoff regarding their endorsement of item 9. This demonstrates the close association of clinically relevant psychic distress and SI also reported in other studies [10, 17, 42]. The more distress a patient experiences, the more frequent are suicidal thoughts. For example, in our study, all patients that reported having suicidal thoughts almost every day were classified as being clinically distressed in all distress screening instruments, except for one case in the FBK-R23. Among the different factors constituting psychosocial distress, hopelessness and loss of meaning are recurrent themes in many studies that investigated the connection between psychosocial distress and SI [30, 31]. We found not only a correlation between SI and hopelessness but also associations between being clinically relevant distressed and hopelessness, independently of the screening instrument. Interestingly, hopelessness as well as loss of meaning are also two constitutive factors of the demoralization syndrome [12, 43, 44], which according to recent research seems better able to capture reasons for distress in cancer patients than psychiatric disorders [10, 30, 45]. For example, mood and anxiety disorders seem to show similar prevalence rates in cancer patients (2.6–12.5%) as in the general population [46], whereas about 20% of cancer patients show clinically relevant symptoms of demoralization [13]. Demoralization is associated not only with psychosocial distress but also with SI and seems better able to explain distress in cancer patients than psychiatric disorders [10, 31, 45]. Furthermore, as Robinson et al. [44] have shown demoralization is negatively associated with patients will to live, making the demoralization construct uniquely suited to identify cancer patients at risk for suicide. This is corroborated by the fact that concerning SI, demoralization has explanatory power beyond that of mental disorders [45]. Fang et al. [10] investigated the role of demoralization in the interplay of distress and SI. They tested mediation models with either demoralization or depression as mediators and found a stronger effect size for demoralization as mediator. In clinical practice, it is often recognized that the description of distress in cancer patients in terms of mental disorders is highly problematic. Cancer patients often do not fulfill the required criteria for a diagnosis, because the diagnostic criteria were not developed to accommodate the specific situations of somatically ill patients. For example, in the case of anxiety disorders, many cancer patients report severe anxiety, and regarding the perceived existential danger in which patients are put by their diagnosis, these fears are of course not irrational. Therefore, a screening tool that is not only able to capture distress on a general level but would also be able to identify patients at risk for suicidality could be the first step in prevention. This is especially pertinent because demoralization predicts a breakdown of coping and thereby a loss of perceived self-efficacy, which, for example, is also an important assumption in the cry-of-pain-model [47].

### Additional factors associated with suicidal ideation

Overall, in our study, different dimensions of distress either self- or externally assessed were associated with SI in cancer patients. Externally rated sadness and deficits in activities of daily life were most strongly associated with self-rated SI. This would suggest that those factors were most obvious to the raters and therefore warrant further investigation. Similarly, self-assessed psycho-somatic afflictions, social restrictions, and restrictions in daily life were most strongly associated with SI. This suggest that similar dimensions of distress, regardless which method of assessment was used, are indicative of SI. In order to educate health care workers to be more attentive not only to verbal but also non-verbal cues of distress, it seems important to identify especially pertinent cues and elaborate reliably methods of detection [1]. Finally, we also found associations between SI and being in a relationship, having children, and current functional status. It can be assumed that those indicate a protective factor, which is in line with what other studies have found [10, 48]. Being in a stable relationship is a source of social support and affords cancer patients with additional resources on which to rely in times of crisis. It is also possible that it helps in strengthening a sense of belonging and purpose, factors negatively affected by demoralization [43], and thereby has a preventative effect. Overall, research on preventative factors is still in its infancy and it would be recommendable to expand it.

## Limitations

There are some factors that are limiting to this study. First, there is the fact that the study sample is a non-representative, mono-centric convenience sample, which has a limiting influence on the overall generalizability of the results. A further problem with convenience samples is introducing bias by self-selection. This might be reflected in the fact that in comparison to the patients excluded, the study sample is younger on average. The reason for this is not clear, as this was the only systematic deviation between included and excluded patients. It could be hypothesized that younger patients are more willing to participate in psycho-oncological studies, because they feel less stigmatized by utilizing psycho-oncological help. Concerning the assessment SI with item 9 of the PHQ-9, it has to be remarked that item 9 confounds self-harm and suicidal thoughts. It is also not possible to differentiate between active and passive suicidal thoughts or thoughts with the implicit intention of regaining a measure of control. Therefore, it seems likely that with this method of assessment a higher rate of false positives will be acquired than with more precise assessment methods. The exploratory nature of the study required a large number of statistical tests which is associated with an increased probability of alpha error accumulation. Concerning the self-report measures, it must be kept in mind that social desirability, as well as exaggeration and attenuation effects, might be a relevant source of bias. Nevertheless, all instruments are widely used in clinical context and have proved validity and reliability. Reliability and validity of the external assessment instrument (PO-Bado-SF) relies on trained raters. Therefore, all interviewers underwent extensive training, including trial interviews and consistency exercises.

## Conclusion

Suicidal ideation is common in cancer patients. There exists a direct relation between factors of psychosocial distress, the demoralization syndrome, and SI. Suicidal thoughts and behaviors are among the most important indicators for suicidality and completed suicides. It is therefore important to be able to identify at risk patients and provide them with adequate care. Screening for suicidality is the first step of prevention. It is therefore necessary to critically examine the currently used screening methods regarding their ability to also identify suicidal patients. Further research in this direction is required to better understand the complex interplay of distress and suicidality. The demoralization construct [43] as well as theory-based research in suicidology, for example the cry-of-pain-model [47], seems to lay a promising groundwork onto which further investigations can be based.

## Supplementary Information

Below is the link to the electronic supplementary material.

Supplementary file1 (DOCX 18 KB)

## Data Availability

The data that support the findings of this study are available on request from the corresponding author. The data are not publicly available due to privacy or ethical restrictions.

## Code availability

N/A.

## References

[1] Henson KE, Brock R, Charnock J, Wickramasinghe B, Will O, Pitman A (2019). Risk of suicide after cancer diagnosis in England. JAMA Psychiat.

[2] Zaorsky NG, Zhang Y, Tuanquin L, Bluethmann SM, Park HS, Chinchilli VM (2019). Suicide among cancer patients. Nat Commun.

[3] Rahouma M, Kamel M, Abouarab A, Eldessouki I, Nasar A, Harrison S, Lee B, Shostak E, Morris J, Stiles B, Altorki NK, Port JL (2018). Lung cancer patients have the highest malignancy-associated suicide rate in USA: a population-based analysis. Ecancermedicalscience.

[4] Senf B, Fettel J, Gog C, Keller M, Maiwurm P (2020) Suizidalität in der Onkologie: Herausforderung und Chance. Hessisches Ärzteblatt 4/2020:240–245. https://www.laekh.de/heftarchiv/ausgabe/artikel/2020/april-2020/suizidalitaet-in-der-onkologie. Accessed 18 Feb 2021

[5] Klonsky ED, May AM, Saffer BY (2016). Suicide, suicide attempts, and suicidal ideation. Annu Rev Clin Psychol.

[6] Brown GK, Steer RA, Henriques GR, Beck AT (2005). The internal struggle between the wish to die and the wish to live: a risk factor for suicide. Am J Psychiatry.

[7] Walker J, Hansen CH, Hodges L, Thekkumpurath P, O’Connor M, Sharma N, Kleiboer A, Murray G, Kroenke K, Sharpe M (2010). Screening for suicidality in cancer patients using Item 9 of the nine-item patient health questionnaire; does the item score predict who requires further assessment?. Gen Hosp Psychiatry.

[8] Walker J, Hansen CH, Butcher I, Sharma N, Wall L, Murray G, Sharpe M (2011). Thoughts of death and suicide reported by cancer patients who endorsed the “suicidal thoughts” item of the PHQ-9 during routine screening for depression. Psychosomatics.

[9] Brinkman TM, Zhang N, Recklitis CJ, Kimberg C, Zeltzer LK, Muriel AC, Stovall M, Srivastava DK, Sklar CA, Robison LL, Krull KR (2014). Suicide ideation and associated mortality in adult survivors of childhood cancer. Cancer.

[10] Fang CK, Chang MC, Chen PJ, Lin CC, Chen GS, Lin J, Hsieh RK, Chang YF, Chen HW, Wu CL, Lin KC, Chiu YJ, Li YC (2014). A correlational study of suicidal ideation with psychological distress, depression, and demoralization in patients with cancer. Support Care Cancer.

[11] Riba MB, Donovan KA, Andersen B, Braun I, Breitbart WS, Brewer BW, Buchmann LO, Clark MM, Collins M, Corbett C, Fleishman S, Garcia S, Greenberg DB, Handzo RGF, Hoofring L, Huang CH, Lally R, Martin S, McGuffey L, Mitchell W, Morrison LJ, Pailler M, Palesh O, Parnes F, Pazar JP, Ralston L, Salman J, Shannon-Dudley MM, Valentine AD, McMillian NR, Darlow SD (2019). Distress management, version 3.2019, NCCN clinical practice guidelines in oncology. J Natl Compr Cancer Netw.

[12] Grassi L, Nanni MG (2016). Demoralization syndrome: new insights in psychosocial cancer care. Cancer.

[13] Robinson S, Kissane DW, Brooker J, Burney S (2015). A systematic review of the demoralization syndrome in individuals with progressive disease and cancer: a decade of research. J Pain Symptom Manage.

[14] Litster B, Bernstein CN, Graff LA, Walker JR, Fisk JD, Patten SB, Bolton JM, Sareen J, El-Gabalawy R, Marrie RA (2018). Validation of the PHQ-9 for Suicidal ideation in persons with inflammatory bowel disease. Inflamm Bowel Dis.

[15] Louzon SA, Bossarte R, McCarthy JF, Katz IR (2016). Does suicidal ideation as measured by the PHQ-9 predict suicide among VA patients?. Psychiatr Serv.

[16] Walker J, Waters RA, Murray G, Swanson H, Hibberd CJ, Rush RW, Storey DJ, Strong VA, Fallon MT, Wall LR, Sharpe M (2008). Better off dead: Suicidal thoughts in cancer patients. J Clin Oncol.

[17] Na PJ, Yaramala SR, Kim JA, Kim H, Goes FS, Zandi PP, Vande Voort JL, Sutor B, Croarkin P, Bobo WV (2018). The PHQ-9 Item 9 based screening for suicide risk: a validation study of the Patient Health Questionnaire (PHQ)-9 Item 9 with the Columbia Suicide Severity Rating Scale (C-SSRS). J Affect Disord.

[18] Glazer K, Rootes-Murdy K, van Wert M, Mondimore F, Zandi P (2020). The utility of PHQ-9 and CGI-S in measurement-based care for predicting suicidal ideation and behaviors. J Affect Disord.

[19] Senf B, Grabowski K, Spielmann N, Fettel J (2020). Quality of life and distress assessed with self and external assessment screening tools in patients with hematologic malignancies attending treatment in an acute hospital. Quality of Life Research: An International Journal of Quality of Life Aspects of Treatment, Care and Rehabilitation.

[20] Senf B, Brandt H, Dignass A, Kleinschmidt R, Kaiser J (2010). Psychosocial distress in acute cancer patients assessed with an expert rating scale. Support Care Cancer.

[21] Herschbach P, Book K, Brandl T, Keller M, Marten-Mittag B (2008). The Basic Documentation for Psycho-Oncology (PO-Bado): an expert rating scale for the psychosocial experience of cancer patients. Onkologie.

[22] Marten-Mittag B, Book K, Buchhold B, Dinkel A, Grundobler B, Henrich G, Huber B, Pirker C, Regenberg A, Schickel S, Senf B, Wunsch A, Herschbach P (2015). The basic documentation for psycho-oncology short form (PO-Bado SF)–an expert rating scale for distress screening: development and psychometric properties. Psychooncology.

[23] Herschbach P, Marten-Mittag B, Henrich G (2003). Revision und psychometrische Prüfung des Fragebogen zur Belastung von Krebskranken (FBK-R23). Z Med Psychol.

[24] Manea L, Gilbody S, McMillan D (2012). Optimal cut-off score for diagnosing depression with the Patient Health Questionnaire (PHQ-9): A meta-analysis. CMAJ.

[25] Kroenke K, Spitzer RL (2002). The PHQ-9: a new depression diagnostic and severity measure. Psychiatr Ann.

[26] Cohen J (1988). Statistical power analysis for the behavioral sciences.

[27] Ellis PD (2010) The essential guide to effect sizes. Statistical power, meta-analysis, and the interpretation of research results. Cambridge University Press. 10.1017/CBO9780511761676

[28] Kühnel S, Krebs D (2014) Statistik für die Sozialwissenschaften: Grundlagen, Methoden, Anwendungen (Original-Ausgabe, 7. Auflage Juli 2014). Rororo Rowohlts Enzyklopädie: Vol. 55639. Rowohlt Taschenbuch Verlag, Reinbek bei Hamburg

[29] Zhong BL, Li SH, Lv SY, Tian SL, Liu ZD, Li XB, Zhuang HQ, Tao R, Zhang W, Zhuo CJ (2017). Suicidal ideation among Chinese cancer inpatients of general hospitals: prevalence and correlates. Oncotarget.

[30] Moreno-Montoya J, Palacios-Espinosa X, Gracia-Ruiz J (2017). Association between religion and suicidal behaviors in cancer patients. Revista Colombiana De Psiquiatria.

[31] Bobevski I, Kissane DW, Vehling S, McKenzie DP, Glaesmer H, Mehnert A (2018). Latent class analysis differentiation of adjustment disorder and demoralization, more severe depressive and anxiety disorders, and somatic symptoms in patients with cancer. Psychooncology.

[32] Henry M, Rosberger Z, Bertrand L, Klassen C, Hier M, Zeitouni A, Kost K, Mlynarek A, Richardson K, Black M, MacDonald C, Zhang X, Chartier G, Frenkiel S (2018). Prevalence and risk factors of suicidal ideation among patients with head and neck cancer: longitudinal study. Otolaryngol Head Neck Surg.

[33] Forkmann T, Brähler E, Gauggel S, Glaesmer H (2012). Prevalence of suicidal ideation and related risk factors in the German general population. J Nerv Ment Dis.

[34] Borges G, Nock MK, Haro Abad JM, Hwang I, Sampson NA, Alonso J, Andrade LH, Angermeyer MC, Beautrais A, Bromet E, Bruffaerts R, Girolamo GD, Florescu S, Gureje O, Hu C, Karam EG, Kovess-Masfety V, Lee S, Levinson D, Medina-Mora ME, Ormel J, Posada-Villa J, Sagar R, Tomov T, Uda H, Williams DR, Kessler RC (2010). Twelve-month prevalence of and risk factors for suicide attempts in the World Health Organization World Mental Health Surveys. J Clin Psychiatry.

[35] Kolva E, Hoffecker L, Cox-Martin E (2020). Suicidal ideation in patients with cancer: a systematic review of prevalence, risk factors, intervention and assessment. Palliat Support Care.

[36] Robson A, Scrutton F, Wilkinson L, MacLeod F (2010). The risk of suicide in cancer patients: a review of the literature. Psychooncology.

[37] Ernst M, Brähler E, Wild PS, Jünger C, Faber J, Schneider A, Beutel ME (2020). Risk factors for suicidal ideation in a large, registry-based sample of adult long-term childhood cancer survivors. J Affect Disord.

[38] Calati R, Fang F, Mostofsky E, Shen Q, Di Mattei VE, Garcia-Foncillas J, Baca-Garcia E, Cipriani A, Courtet P (2018). Cancer and suicidal ideation and behaviours: protocol for a systematic review and meta-analysis. BMJ Open.

[39] Senf B, Fettel J, Demmerle C, Maiwurm P (2019). Physicians’ attitudes towards psycho-oncology, perceived barriers, and psychosocial competencies: indicators of successful implementation of adjunctive psycho-oncological care?. Psychooncology.

[40] Ramberg IL, Di Lucca MA, Hadlaczky G (2016). The impact of knowledge of suicide prevention and work experience among clinical staff on attitudes towards working with suicidal patients and suicide prevention. Int J Environ Res Public Health.

[41] Senf B, Maiwurm P, Fettel J (2020). Exposure to suicidality in professionals working with oncology patients: an online survey. Psychooncology.

[42] Munson SO, Cabrera-Sanchez P, Miller SN, Phillips KM (2020). Distress and factors associated with suicidal ideation in veterans living with cancer. Federal Practitioner: For the Health Care Professionals of the VA, DoD, and PHS.

[43] Kissane DW (2014). Demoralization: a life-preserving diagnosis to make for the severely medically ill. J Palliat Care.

[44] Robinson S, Kissane DW, Brooker J, Hempton C, Michael N, Fischer J, Franco M, Sulistio M, Clarke DM, Ozmen M, Burney S (2016). Refinement and revalidation of the demoralization scale: the DS-II-external validity. Cancer.

[45] Vehling S, Kissane DW, Lo C, Glaesmer H, Hartung TJ, Rodin G, Mehnert A (2017). The association of demoralization with mental disorders and suicidal ideation in patients with cancer. Cancer.

[46] Mehnert A, Vehling S, Scheffold K, Ladehoff N, Schön G, Wegscheider K, Heckl U, Weis J, Koch U (2013). Prävalenz von Anpassungsstörung, Akuter und Posttraumatischer Belastungsstörung sowie somatoformen Störungen bei Krebspatienten [Prevalence of adjustment disorder, acute and posttraumatic stress disorders as well as somatoform disorders in cancer patients]. Psychother Psychosom Med Psychol.

[47] Williams JMG (2002). Suicide and attempted suicide.

[48] Akechi T, Okuyama T, Uchida M, Kubota Y, Hasegawa T, Suzuki N, Komatsu H, Kusumoto S, Iida S (2020). Factors associated with suicidal ideation in patients with multiple myeloma. Jpn J Clin Oncol.

