# Supplementary material for: Suicidal ideation, distress, and related factors in a population of cancer patients treated in a general acute hospital
Source: Support Care Cancer. 2021 Jul 29;30(1):487–96. doi: 10.1007/s00520-021-06429-w (PMC8636422; doi:10.1007/s00520-021-06429-w)
Supplement: Supplementary file 1 — Supplementary file1 (DOCX 18 KB) [file 520_2021_6429_MOESM1_ESM.docx]

# Supplementary material

Table S1: Descriptive statistics of psychosocial distress (PO-Bado-SF, PHQ-9, QSC-R23).

|  | *N* | *M* | *SD* |
| --- | --- | --- | --- |
| PO-Bado-SF |  |  |  |
| Fatigue/tiredness | 226 | 1.2 | 1.1 |
| Mood-swings | 226 | 1.5 | 1.1 |
| Functional limitations in daily livings | 226 | 1.1 | 1.2 |
| Anxiety/worries/tension | 226 | 2.1 | 1.0 |
| Grief/depression | 226 | 1.6 | 1.1 |
| Other problems, e. g. social or family problems | 95 | 1.1 | 1.1 |
| PHQ-9 |  |  |  |
| Little interest or pleasure in doing things | 226 | 0.8 | 0.9 |
| Feeling down, depressed, or hopeless | 226 | 0.7 | 0.8 |
| Trouble falling or staying asleep, or sleeping too much | 226 | 1.2 | 1.1 |
| Feeling tired or having little energy | 226 | 1.1 | 1.0 |
| Poor appetite or overeating | 226 | 0.9 | 1.0 |
| Feeling bad about yourself – or that you are a failure or have let yourself or your family down | 226 | 0.1 | 0.5 |
| Trouble concentrating on things, such as reading the newspaper or watching television | 226 | 0.7 | 0.8 |
| Moving or speaking so slowly that other people could have noticed? Or the opposite – being so fidgety or restless that you have been that you have been moving around a lot more than usual | 226 | 0.4 | 0.8 |
| Thoughts that you would be better off dead or of hurting yourself in some way | 226 | 0.2 | 0.5 |
| QSC-R23 |  |  |  |
| Feeling tired and weak | 226 | 1.6 | 1.7 |
| Suffering pain due to surgery | 226 | 0.9 | 1.4 |
| Feeling unconfident in relationships with other people | 226 | 0.3 | 0.8 |
| Suffering pain due to unknown causes | 226 | 0.8 | 1.5 |
| Being afraid of disease progression | 226 | 2.3 | 1.8 |
| Other people react inconsiderately/ unsympathetically | 226 | 0.3 | 0.8 |
| Body care has become difficult | 226 | 0.7 | 1.3 |
| Being afraid of developing pain | 226 | 1.6 | 1.7 |
| Having the feeling of being less value for other people | 226 | 0.3 | 0.8 |
| Being afraid of having to go to the hospital again | 226 | 1.5 | 1.7 |
| Feeling physically imperfect | 226 | 0.9 | 1.5 |
| Not being able to follow one’s hobbies | 226 | 1.6 | 1.8 |
| Having trouble sleeping | 226 | 1.5 | 1.7 |
| Being afraid of not being able to work anymore | 226 | 1.1 | 1.7 |
| Feeling not well informed about illness/treatment | 226 | 0.4 | 1.1 |
| Feeling often tense and nervous | 226 | 1.3 | 1.4 |
| Having sex less frequently | 226 | 1.4 | 1.8 |
| Feeling not adequately informed about social support | 226 | 0.9 | 1.6 |
| Difficulty in talking with the family | 226 | 0.3 | 0.9 |
| Going out less | 226 | 1.1 | 1.7 |
| Different information from different doctors | 226 | 0.6 | 1.4 |
| Having too few opportunities to talk about emotional problems | 226 | 0.6 | 1.2 |
| Difficulty for partner to empathise my situation | 226 | 0.5 | 1.1 |

Table S2: Correlation (Kendall-Tau-b) between PHQ-9 item 9 („Thoughts that you would be better off dead or of hurting yourself in some way“) and the items of the QSC-R23.

|  | PHQ-9 item 9 |
| --- | --- |
| Feeling tired and weak | 0.308** ^a^ |
| Suffering pain due to surgery | 0.110 |
| Feeling unconfident in relationships with other people | 0.175** |
| Suffering pain due to unknown causes | 0.154* |
| Being afraid of disease progression | 0.123* |
| Other people react inconsiderately/ unsympathetically | 0.175** |
| Body care has become difficult | 0.209** |
| Being afraid of developing pain | 0.186** |
| Having the feeling of being less value for other people | 0.239** |
| Being afraid of having to go to the hospital again | 0.152* |
| Feeling physically imperfect | 0.233** |
| Not being able to follow one’s hobbies | 0.230** |
| Having trouble sleeping | 0.219** |
| Being afraid of not being able to work anymore | 0.151* |
| Feeling not well informed about illness/treatment | 0.203** |
| Feeling often tense and nervous | 0.246** |
| Having sex less frequently | 0.077 |
| Feeling not adequately informed about social support | 0.169** |
| Difficulty in talking with the family | 0.187** |
| Going out less | 0.216** |
| Different information from different doctors | 0.132* |
| Having too few opportunities to talk about emotional problems | 0.281** |
| Difficulty for partner to empathise my situation | 0.246** |

^a^ Classification of correlation coefficients (based on Pearson's r coefficient and adopted analogously for Kendall-Tau-b): No correlation: 0.00<*r_τ_*<0.05, low correlation: 0.05<*r_τ_*<0.20, medium correlation: 0.20<*r_τ_*<0.50, high correlation: 0.50<*r_τ_*<0.70, very high correlation: *r_τ_*>0.70 [28].

* *p*<0.05; ** *p*<0.01.

Table S3: Associations between „Thoughts that you would be better off dead or of hurting yourself in some way“ and sociodemographic information.

|  | PHQ-9 item 9 |
| --- | --- |
| Age | - 0.051 ^a b^ |
| Sex | *χ²*=3.99; V=0.133 ^c^ |
| Steady relationship | *χ²*=8.12; V=0.190* |
| Children | *χ²*=10.10; V=0.211* |
| Job situation | *χ²*=6.83; V=0.101 |
| Metastases | *χ²*=1.21; V=0.052 |
| Current disease status | *χ²*=6.80; V=0.124 |
| Psychopharmaceuticals/opiates | *χ²*=4.46; V=0.099 |
| Current function status | *χ²*=19.05; V=0.230* |

^a^ The correlation between „Thoughts that you would be better off dead or of hurting yourself in some way“ and age is the Kendall-Tau-b correlation coefficient.

^b^ Classification of correlation coefficients (based on Pearson's r coefficient and adopted analogously for Kendall-Tau-b): No correlation: 0.00<*r_τ_*<0.05, low correlation: 0.05<*r_τ_*<0.20, medium correlation: 0.20<*r_τ_*<0.50, high correlation: 0.50<*r_τ_*<0.70, very high correlation: *r_τ_*>0.70 [28].

^c^ The other measures of association are chi-square (*χ²*) and Cramér's V. Classification for Cramér's V: small effect: V=0.1, medium effect: V=0.3, large effect: V=0.5 [26, 27].

* *p*<0.05; ** *p*<0.01.
